# Electronic Games for Facilitating Social Interaction Between Parents With Cancer and Their Children During Hospitalization: Interdisciplinary Game Development

**DOI:** 10.2196/16029

**Published:** 2021-01-21

**Authors:** Karin Piil, Helle Holm Gyldenvang, Jeppe Kilberg Møller, Tine Kjoelsen, Jesper Juul, Helle Pappot

**Affiliations:** 1 Department of Oncology Copenhagen University Hospital (Rigshospitalet) Copenhagen Ø Denmark; 2 Department of Public Health Aarhus University Aarhus Denmark; 3 Department of Visual Design Schools of Architecture, Design and Conservation The Royal Danish Academy Copenhagen K Denmark; 4 Department of Clinical Medicine Copenhagen University Copenhagen Denmark

**Keywords:** cancer patients, children, adolescents, social relation, emotional well-being, gamification, relatives, visual design, serious games

## Abstract

**Background:**

Most cancer treatments today take place in outpatient clinics; however, it might be necessary for some patients to be admitted to hospital departments due to severe side effects or complications. In such situations, support from family and social relations can be crucial for the patients’ emotional well-being. Many young adolescents and children whose parents have cancer describe how they are not seen, heard, or listened to as the worried relatives they are. Within the intensive care unit, it has been recommended that early supportive interventions are tailored to include children of the intensive care patient; a similar approach might be relevant in the oncological setting. To our knowledge, no studies have explored how to involve young relatives who are visiting their parent at an oncological department. Recently, a framework for developing theory-driven, evidence-based serious games for health has been suggested. Such a process would include stakeholders from various disciplines, who only work toward one specific solution. However, it is possible that bringing together different disciplines, such as design, art, and health care, would allow a broader perspective, resulting in improved solutions.

**Objective:**

This study aims to develop tools to enhance the social interaction between a parent with cancer and their child when the child visits the parent in the hospital.

**Methods:**

In total, 4 groups of design students within the Visual Design program were tasked with developing games addressing the objective of strengthening relations in situ during treatment. To support their work, the applied methods included professional lectures, user studies, and visual communication (phase I); interviews with the relevant clinicians at the hospital (phase II), co-creative workshops with feedback (phase III), and evaluation sessions with selected populations (phase IV). The activities in the 4 phases were predefined. This modified user design had the child (aged 4-18 years) of a parent with cancer as its primary user.

**Results:**

Overall, 4 different games were designed based on the same information. All games had the ability to make adults with cancer and their children interact on a common electronic platform with a joint goal. However, the interaction, theme, and graphical expression differed between the games, suggesting that this is a wide and fertile field to explore.

**Conclusions:**

Playing a game can be an efficient way to create social interaction between a parent with cancer and a child or an adolescent, potentially improving the difficult social and psychological relations between them. The study showed that the development of serious games can be highly dependent on the designers involved and the processes used. This must be considered when a hospital aims to develop multiple games for different purposes.

## Introduction

Patients diagnosed with cancer often undergo aggressive oncological treatment mainly provided by outpatient clinics at hospitals [1]. The patients experience a wide range of changing symptoms that occur due to the cancer itself as well as side effects from the treatment [2]. Sometimes, there are complications, resulting in a potentially life-threatening situation for the patient, requiring admission to the hospital. The two main disadvantages of such situations are related. First, the patients may suffer due to being away from their family [3]. Second, patients find themselves in a distressing situation and are left alone with their concerns and worries. In these situations, patients benefit from receiving emotional and social support from their close relatives. Social support includes personal, informal advice, and can provide strength that helps individuals initiate and sustain self-management activities. Existing literature has identified a correlation between patients’ perceived degree of social support and quality of life [4]. Further, children and adolescents with a parent with cancer have noted that they are not seen, heard, and listened to as the worried relative they are [5]. Few studies have explored the experiences of children and adolescents across a parent’s illness trajectory; however, a recent meta-synthesis recommended early supportive interventions tailored to include the children of intensive care patients [6]. To our knowledge, no studies have explored how to include children who are visiting their parent at an oncological department. Still, there is a general acknowledgment that health care professionals need to reflect on how to approach children and young adolescents at the hospital when they are visiting a parent [5,7]. Gamification, serious gaming, and edutainment have the potential to establish enjoyable and motivating experiences when gaming elements are introduced in a nongaming context [8]. The notion of gamification and serious gaming is traditionally used to increase adherence to medication regimens, promote health education for patients, and offer apps for chronic disease management [9]. Video games and serious gaming have the ability to enchant and engage patients and their children in a different “world” and thus give them a collective short break from the otherwise rather serious context they are in together. We are not aware of other studies aimed at facilitating social and joyful interaction between a parent with cancer and their children through gamification during hospitalization in an oncological department. However, virtual emotional authenticity and feelings of empathy may be able to help patients with cancer frame the often traumatizing experiences in more positive terms [10]. The development of serious games for such purposes is, as in eHealth generally, based on scientific and design foundations [11]. However, the product developed for a given purpose might be dependent on the software provider, the processes, or the specific designer asked to address specific needs. By bringing together different disciplines and acknowledging the diversity in art and design, more solutions might be designed for the same purpose [12]. This study aims to develop tools to enhance social interaction between a parent with cancer and their children when the children visit the parent at the hospital.

## Methods

### Overview

This study is based on an interdisciplinary co-creation process with clinical oncology specialists from Copenhagen University Hospital (KP, HG, and HP), gaming and visual design experts from The Royal Danish Academy of Architecture, Design, and Conservation (TK, JK, and JJ), and second-year bachelor’s degree students at the Academy. The study did not need ethical approval. For this study, over 3 months, 4 different groups of design students within the Visual Design program were tasked with developing games addressing the objectives of the field of design and focusing on strengthening relations in situ during treatment. This modified user-centered design had the child (aged 4-18 years) of a parent with cancer as its primary user. Target age groups were defined in the initial phases of the project based on statistical information from the department and the already established research focus of next of kin and young adults as relatives, as well as by established researchers from the field of developmental psychology and the complexity of the developed games [12]. We approached the target groups through the lens of media use, where a concrete game is part of the broader media ecology, meaning that material from a television series can cross into a video game, and a video game can cross into children’s outdoor analog play [13]. This has a specificity that exceeds the level of detail offered by developmental psychology, and the guiding principle for design was therefore observations of the actual game and media use by children in the respective age brackets [13]. The activities in the 4 phases were structured and predefined.

### Phase I

The clinical specialists presented the clinical settings and environment of the oncological department (eg, standard cancer disease and treatment trajectories) to the students, as well as the context of the study aim and ethical issues. The students signed a confidentiality agreement. Discussions on how to establish and strengthen social and joyful togetherness between the admitted parent with cancer and their children were facilitated. There was an underlying consideration of how to nurture the experience of social support that may play an important role in a multifaceted approach to preventing complicated grief among relatives. The hospital and its staff were defined as customers in the development process, and the field of developmental psychology was researched throughout the process [13].

### Phase II

The students interviewed nurses (n=4) who were selected to be informers due to their extensive experiences with patients with cancer and their relatives.

### Phase III

Students listened to the story of a bereaved child from The Danish National Center for Grief. Students were given a lecture by a researcher with a special interest in and knowledge of children as relatives of parents with cancer [14]. Students could ask questions of and engage in discussions with both lecturers.

### Phase IV

The students presented their initial prototypes and received feedback from the multidisciplinary team. Acceptability testing was performed in an external population; a class of healthy children from the same age group was used for this purpose. Adjustments were incorporated ahead of an official presentation at the hospital and visitors at the prototype exhibition were randomly included in the usability test. Hence, the prototypes were tested for usability using this small, selected population via surveys. Tests of the prototypes with hospitalized parents with cancer and their children were not performed due to ethical considerations.

## Results

In phases I-III, the facts used for the design process were documented in written reports by the 4 working groups. Information from interdisciplinary stakeholders such as patients, nurses, research specialists, clinical oncological specialists, and gaming and visual design experts was included as a design foundation for all reports. In phase IV, 4 independent prototype games were developed (Table 1). Each game included an acceptance test using nontarget populations.

**Table 1:** Prototypes of the games.

Game title	Theme	Target age group (years)	Element of interaction	How the game aims to create social interaction	Number of players
Fun and Cozy Socks	Good mood gaming	15-18	Sample of social and interactive guessing minigame	Establishes a feel-good atmosphere with competition and laughs	2-3
The Witch	Magic team building game	4-8	Cooperative multiplayer game, book, and electronic game	Provides a fun and enjoyable activity that builds trust between the players	2
Kurt, the Hospital Cat	Augmented reality	6-12	The interactive experience of a real-world environment with a search for items in the physical surroundings	Adds information and meaning to the hospital surroundings	1 (includes the possibility of interacting with a parent in search for items)
Bloom	Growing a garden and bringing it to life	4-18	Customization/minigames	Makes the players feel good and is enjoyable. Shows that you care for a person by growing flowers and taking care of them.	1 player on a social platform

In general, the prototypes were highly accepted by age-equivalent testing groups. A usability test among random visitors at the parallel presentation of all 4 solutions included patients (n=6), health care professionals (n=10), and young adults (n=3). The survey results included good ratings for “interesting game,” “user friendly,” and “willingness to use” in a hospital setting. In general, all solutions were positively evaluated, reflecting a fundamental interest in playing games to experience joyful moments. However, the older population (aged >70 years) seemed to have reduced digital competencies and a lower willingness to use such games.

**Figure 1:**
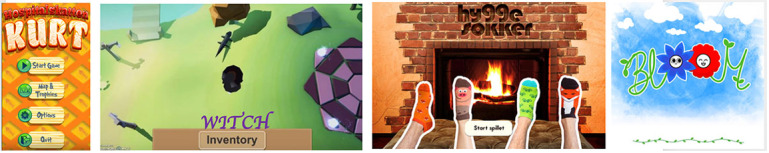
Front pages for game prototypes.

The prototypes (Figure 1) were tested on a small, selected population via a paper-based survey. Physical contact testing was not possible due to ethical concerns.

## Discussion

### Principal Findings

Based on the same information and evaluation, 4 different working groups skilled in visual design developed games with different themes and interactions, for different target age groups, to solve the same challenge. This study shows that gamification has the potential to solve newly revealed needs within health care, such as improving interactions between a parent with cancer and their child. This study suggests that several types of designs can solve the same problem, but also underscores that solutions are highly dependent on the software developer and designer. These factors should be considered when, for example, a hospital aims to develop multiple games for different purposes, or when the best game for a specific purpose is requested.

It has previously been described how patient and family involvement is valuable for health [15]; however, little is known about the involvement of children as relatives. In the adult setting, eHealth tools have been developed for adult relatives [16] to improve their psychological well-being, but not to improve their interactions with the patient. It is known that web-based tools and games can be used to improve adolescents' and young adults’ adherence to vaccination programs and therapy [17,18]. It seems that eHealth tools can improve psychological outcomes [19]. It might be argued that the development of serious games for health should be based on a framework suggested by Verschueren et al [11]. However, to improve the quality of serious games for health, this study tries to encourage interdisciplinary cooperation between art and health science experts to elicit a greater number of suggestions of game-based solutions for the same purpose, followed by a selection of the best feasible solution for a specific need considering the different themes and graphic design elements of the developed games.

### Limitations

A limitation of this study might be that the tools were developed by students; however, they were supervised by experts in gamification and evaluated and given continuous feedback during the process by user design and gamification experts.

A modified concept of user-centered design was applied to this tool developing process, where experiences from one young relative and other stakeholders were the basis for the development. However, this one representative was used to represent a group of children and young relatives of patients with cancer.

All 4 working groups performed an acceptance test of their product in a nonhospital environment (eg, a day care facility or school). The usability test involved the parallel testing of all 4 tools in a hospital environment and included children/young people, patients/relatives, and hospital staff. None of these tests were performed with a larger group of children who are relatives of patients with cancer; thus, the generalizability of the findings must be further explored.

### Conclusion

A game can be an efficient method for fostering social interaction between a parent with cancer and a child or an adolescent, and games likely have the power to improve the difficult social and psychological relations between parents and children in this context. When creating games for specific purposes such as this, it must be remembered that games designed based on the same assignment might use different themes and graphic design elements, indicating that the development of serious games may be highly dependent on the designer involved. The described games remain to be investigated more thoroughly regarding their feasibility and efficacy in the context of qualitative oncological research. If shown to be feasible, implementation among patients with cancer and their relatives will be carried out.
